# Assessing knowledge and awareness of Food and Drug Interactions among nutrition sciences students: Implications for education and clinical practice

**DOI:** 10.1177/02601060241263409

**Published:** 2024-07-23

**Authors:** Sofia Beirão, João G. Costa, Cíntia Ferreira-Pêgo

**Affiliations:** 1 Escola de Ciências e Tecnologias da Saúde, Universidade Lusófona, Lisboa, Portugal; 2 CBIOS Lusófona's Research Center for Biosciences and Health Technologies, Lisbon, Portugal

**Keywords:** Knowledge, Food–Drug Interactions, nutritionists, nutrition sciences students, public health

## Abstract

**Background:**

Chronic diseases and polymedication increase the risk of food–drug interactions (FDIs) among the population, negatively impacting health. Nutritionists, as responsible for dietary planning, have a key role in preventing these events.

**Aim:**

To assess the knowledge about FDIs among a sample of Nutrition Sciences Bachelor students.

**Methods:**

A descriptive cross-sectional observational study was conducted, involving 44 students from the 3^rd^ and 4^th^ academic years of different Portuguese universities during the 2023/2024 academic year. Participants completed a self-reported questionnaire, covering general and specific FDIs knowledge, academic background, and perceptions regarding FDIs importance and training adequacy.

**Results:**

Results revealed a general lack of FDIs knowledge among participants, particularly in identifying specific interactions and appropriate dietary management. While half of the students reported exposure to a subject dedicated to FDIs, only 18.18% considered the knowledge acquired sufficient. Nearly all participants (93.18%) expressed the need for further training in FDIs during their undergraduate course. Furthermore, none of the participants had received additional training or attended workshops on FDIs. Specifically, participants struggled to identify appropriate dietary choices in conjunction with certain medications. Moreover, only a minority of participants demonstrated awareness of the ideal timing for medication intake relative to food consumption. Despite these knowledge gaps, participants recognized the importance of FDIs knowledge for future clinical practice.

**Conclusion:**

Bridging these knowledge gaps through targeted educational interventions and interdisciplinary collaboration is essential to ensure future nutrition professionals are equipped to address the complex challenges posed by FDIs in professional practice.

## Introduction

Nowadays, chronic diseases are becoming increasingly prevalent, posing a major public health challenge on a global scale. This problem is rising rapidly due to population growth and an aging society (Centers for Disease Control and Prevention, 2024). As a consequence, polymedicated patients are also increasing (Ramírez-Duque et al., 2014), having a higher risk of food–drug interactions (FDIs) (Grabowsky, 2013). Elderly patients due to different chronic conditions such as diabetes and cardiovascular diseases are particularly susceptible to FDIs (Osuala et al., 2022). FDIs can be therefore defined as a change in the pharmacokinetic and/or pharmacodynamic properties of a drug caused by one or more foods (Bushra et al., 2011; Genser, 2008; Ziani, 2022). Considering pharmacokinetic changes, foods can alter the effect of a drug by interfering with its absorption, distribution, metabolism, and excretion (Choi & Ko, 2017; Genser, 2008; Yamreudeewong et al., 1995). Regarding pharmacodynamic modifications, foods can also affect the bioavailability of the drug, by competing, and changing the mode of action or the final biological target. Therefore, FDIs may result in a decrease or increase in the effectiveness of a drug in the body or lead to situations of toxicity or ineffectiveness (Chan, 2002).

Thus, the combination of food and drugs can lead to undesirable interactions that may affect therapeutic outcomes (Abdollahi et al., 2018). Moreover, FDIs can also cause unnecessary adverse reactions, increasing disease morbidity and even leading to patients’ hospitalization (McCabe, 2004). FDIs can occur in ambulatory patients, but also in hospitalized patients who demonstrates the importance and need for the prevention of FDIs to achieve better treatment goals (Bushra et al., 2011; McCabe, 2004).

It is recognized and well-established that all healthcare professionals have an important role to play in identifying these interactions (Deng et al., 2017; Hughes et al., 2002). The Joint Commission on Accreditation of Hospitals encourages healthcare professionals, particularly pharmacists, and nutritionists, to monitor FDIs in hospitalized patients, and to advise them on the subject when they are discharged from the hospital (Bertrand et al., 2014; Unroe et al., 2010). In this sense and given the fundamental role of nutritionists in the design of dietary plans, they must have increased knowledge of FDIs. This competence will enable them to optimize the therapeutic process of their patients without jeopardizing their lives and their treatment (Deng et al., 2017).

Nevertheless, scientific evidence shows that there are significant knowledge gaps among different healthcare professionals (Coelho & Horta, 2021; Colet et al., 2016; Lombardo & Eserian, 2014; Osuala et al., 2021; Silva et al., 2013; Zawiah et al., 2020). Therefore, considering that nutrition students are training to become future nutritionists, it is important to evaluate their understanding of this topic and discuss the relevance of this matter in academic curricular programs.

Hence, this work intends to evaluate knowledge of nutrition sciences and nutrition and dietetics students on FDIs and to assess its importance given to the content covered during their academic curricular programs, and if they believe it to be sufficient to ensure safe clinical practice in their professional practice as future nutritionists.

## Materials and methods

### Study sample and design

The present study consisted of a descriptive cross-sectional observational study, which included a self-reported questionnaire specifically designed to access FDIs knowledge in Nutrition Sciences students.

The sample studied was conveniently selected from nutrition sciences students from 3^rd^ and 4^th^ scholar years, mainly enrolled at the Lusófona University, Lisbon and Coimbra School of Health Technology, and Egas Moniz School of Health & Science, during the 2023/2024 academic year. A total of 44 students participated in this analysis.

Participation in this study was completely voluntary and anonymous and all responses were treated confidentially ensuring the ethical principles of research and good clinical practices. All individuals agreed to participate in the study before data collection through informed written consent. The main project was approved by the Ethics Committee of the School of Sciences and Health Technologies from Lusófona University (CE.ECTS/P09-24).

### Data collection tools and procedures

Data was collected between 15 January 2024 and 8 March 2024, using an online questionnaire disseminated through online personal and institutional platforms and social networks (i.e. LinkedIn® and Meta® networks), as also sent by email to all the Portuguese universities that teach Nutrition Sciences and Nutrition and Dietetics Bachelor.

The questionnaire included five sections: the first was dedicated to written free and informed consent. The second part of the questionnaire was related to the general and sociodemographic characteristics of the participants, such as age, sex, nationality, and place of residence. The third part covered the characterization of the student's academic background, including questions about their academic year, the existence of a specific subject dealing with FDIs, the need for further training in this area to increase confidence in the subject, and the importance attached to knowledge of FDIs in the nutritionists’ clinical and professional practice. This question was evaluated from 0 to 5, in which 0 corresponded to “no important” and 5 to “very important.” The fourth and fifth sections of the questionnaire were based on a previously validated questionnaire (Benni et al., 2012), with some adaptations to the Portuguese context, and related to the assessment of general and specific FDIs knowledge, respectively.

### Statistical analysis

A full descriptive analysis of the results was performed, using Jamovi® statistical software. Data were expressed as mean and standard deviation for continuous variables, and as absolute frequencies (n) and relative frequencies (percentages, %) for categorical variables. A sample size estimation was performed, taking into consideration a confidence interval of 85%, a margin of error of 5%, and a population portion of 15% of 75 individuals, which was the number of individuals studying the 3^rd^ and 4^th^ academic courses of Nutrition Sciences bachelor during the 2023/2024 school year. The final sample needed to include 44 individuals. All statistical tests were two-tailed, and the significance level was set at *p* < 0.05.

## Results

Of the 44 participants included in the present study, approximately 77% were female showing a general mean age of 24.23 years old, being over 80% residents in the Lisbon Metropolitan area. Most of the Nutrition Sciences Bachelor students attended the 3^rd^ curricular year at Lusófona University (Table 1).

**Table 1.** General characteristics of the study sample.

	Total sample (n = 44)
Sex, % (n)	
Female	77.27 (34)
Male	22.73 (10)
Age, years	24.23 ± 7.37
Area of residence, % (n)	
Algarve	2.27 (1)
Lisbon Metropolitan Area	81.82 (36)
Centre	13.64 (6)
Autonomous Region of the Azores	2.27 (1)
Institution of study, % (n)	
Lisbon School of Health Technology	4.55 (2)
Coimbra School of Health Technology	2.27 (1)
Egas Moniz School of Health & Science	2.27 (1)
Lusófona University	90.91 (40)
Curricular year, % (n)	
3^rd^ year	59.09 (26)
4^th^ year	40.91 (18)

Data expressed as means ± SD or percentages (n) for continuous or categorical variables, respectively.

Regarding the educational characterization of the sample (Table 2), half of the participants reported having a subject dedicated exclusively to the study of FDIs. However, only 18.18% of the students considered that the knowledge acquired was sufficient, and almost the total of the sample (93.18%) felt that should have received more training on FDIs during their undergraduate course. It was also observed that none of the participants attended any further or complementary training courses or workshops on FDIs and the majority used scientific articles to research the subject. On a scale from 0 to 5, the importance of having knowledge about FDIs in the clinical practice of nutritionists was ranked 4.50 by the Nutrition Sciences Students inquired.

**Table 2.** Educational characteristics of the study sample.

	Total sample (n = 44)
Had an exclusive discipline about FDIs during the bachelor, % (n)	50.00 (22)
Considering the knowledge acquired during the bachelor sufficient, % (n)	18.18 (8)
Considering should receive more information about FDIs during their Bachelor's, % (n)	93.18 (41)
Enrolled complementary training about FDIs, % (n)	0.00 (0)
Sources of information used to search about FDIs, % (n)	
Websites	34.09 (15)
Scientific articles	54.55 (24)
Drug package leaflets	11.36 (5)
What is the importance attributed to FDIs in the Clinical Practice of the Nutritionist?^a^	4.50 (0.95)

Data expressed as percentages (n) or mean (SD) for categorical or continuous variables, respectively. Abbreviations: FDIs, Food–Drug Interactions. ^a^Score ranged from 0 to 5.

Regarding the assessment of general knowledge about FDIs, as shown in Table 3, all the participants agreed that food and drinks can interfere with the therapeutic effect of a drug. However, only 84.09% of the participants were aware that this interference could either heighten its toxicity or reduce its effectiveness. The majority of the sample (95.45%) also recognized that the effects of these interactions are influenced by various factors such as the dosage of the medication, the age of the individual, and their health status. Regarding the correct definition of FDIs, 65.91% of the participants correctly considered that this interaction refers to drugs and diet, iron, vitamin supplements, alcohol, and fruit juices. Concerning the age groups presenting a higher risk of FDIs, 22.73% of the surveyed individuals considered this interaction risk greater in children lower than 1 year old, and 52.27% considered those 60 or more years old. Still on the risk for FDIs, participants considered that cardiovascular (34.09%), followed by oncological (25.00%) and endocrine diseases (25.00%), were the most important health disorders that Nutritionists should have more access to information about. When questioned, in an open way (questions without pre-defined answer options), about the most frequent and the most severe FDIs, Nutrition Sciences Bachelor Students revealed huge difficulty in indicating correct examples of FDIs or even in answering the question. Nevertheless, warfarin with vitamin K-rich foods was the FDI considered both the most frequent (4.55%) and the most severe (20.45%) by the participants. Upon being asked which FDIs they thought were the most severe, about 45.45% of the participants did not seem to have an opinion about this question or had difficulty selecting one of the options. Nevertheless, 20.45% of the individuals attributed alcohol, and another 20.45% warfarin with vitamin K-rich foods as the most severe FDIs.

**Table 3.** General knowledge about Food–Drug Interactions (FDIs).

	Total sample (n = 44)
Food and drinks can interfere with the therapeutic effect of drugs, % (n)	100.00 (44)
Food and drinks can affect the therapeutic effect of drugs, increasing toxicity, or decreasing effectiveness, % (n)	84.09 (37)
Age groups presenting a higher risk of FDIs, % (n)	
< 1 year old	22.73 (10)
1–4 years old	18.18 (8)
15–45 years old	2.27 (1)
46–59 years old	4.55 (2)
≥ 60 years old	52.27 (23)
Most important diseases to have access to information about the FDIs, % (n)	
Cardiovascular diseases	34.09 (15)
Oncological diseases	25.00 (11)
Neurodegenerative diseases	15.91 (7)
Endocrine diseases	25.00 (11)
The definition of FDIs refers to the interaction between the drug, % (n)	
Diet	29.55 (13)
Iron and vitamins supplements	4.55 (2)
Diet, iron and vitamins supplements, alcohol, and fruit juices	65.91 (29)
The effects of FDIs are influenced by several factors, such as the dosage of medication, age, and health status, % (n)	95.45 (42)
The intake of food and drinks can interfere with drugs, % (n)	
Absorption	93.18 (41)
Metabolism	86.36 (38)
Distribution	61.36 (27)
Excretion	52.27 (23)
All	40.91 (18)
The most frequent FDI, % (n)	
Warfarin and vitamin K-rich foods	4.55 (2)
Metformin and vitamin B12	2.27 (1)
Iron and vitamin C	2.27 (1)
Milk and antibiotics	2.27 (1)
Did not indicate a valid example of FDI	59.09 (26)
Did not know/ Not answered	29.55 (13)
The most severe FDIs, % (n)	
Warfarin and vitamin K-rich foods	20.45 (9)
MAOI and tyramine-rich foods	9.09 (4)
Metformin and vitamin B12	2.27 (1)
Antidepressants and Grapefruit	2.27 (1)
Did not indicate a valid example of FDIs	20.45 (9)
Did not know/ Not answered	45.45 (20)

Data expressed as percentages (n). Abbreviations: FDIs, food–drug interactions; MAOI, monoamine oxidase inhibitor.

In Table 4, it is possible to observe the specific knowledge about FDIs. The majority of the participants (72.73%) correctly considered that the consumption of coffee, tea, and chocolates should be avoided when taking theophylline. However, it was found that there was a lack of knowledge about the foods that can be consumed in combination with monoamine oxidase inhibitors or antibiotics, since merely 36.36% to 38.64% of the participants correctly identified the appropriate dietary choices in conjunction with these medications, respectively. Additionally, it was observed that only 6.82% of the sample were acquainted with the appropriate meal choices during antifungal therapy. Similarly, 15.91% demonstrated awareness of suitable dietary options while undergoing treatment with drugs like propranolol. Moreover, only 18.18% provided accurate responses regarding foods that may interact with hypothyroidism medication. The three questions about foods interacting with warfarin showed that 47.73% were aware of the need to avoid foods such as broccoli, cauliflower, and chickpeas when taking this drug, and 45.45% were aware of the need to avoid prolonged consumption of garlic and ginger. However, only 34.09% were aware that it was also important to avoid eating foods such as Brussels sprouts and spinach. There was a lack of knowledge among the inquired sample about the ideal time to take medicines taking into consideration food intake, which can affect the drug's bioavailability, effect, or safety. For instance, only 29.55% of the students knew that metformin must be taken during or after meals. However, most of the participants knew that omeprazole should be taken before meals (68.18%) and non-steroidal anti-inflammatory drugs during or after meals (84.09%). Participants’ knowledge increased when they were asked about alcohol consumption and its possible interactions since most participants were aware that alcohol consumption should be avoided when taking paracetamol (86.36%) and antibiotics (84.09%).

**Table 4.** Specific knowledge about Food–Drug Interactions.

	Total sample (n = 44)^a^
The consumption of coffee, tea and chocolates should be avoided when taking Theophylline, % (n)	72.73 (32)
The intake of dairy products should be avoided when taking Tetracyclines, % (n)	36.36 (16)
Cheese, processed meats and beer should be avoided when taking MAOI, % (n)	36.36 (16)
Prolonged intake of garlic and ginger should be avoided while taking Warfarin, % (n)	45.45 (20)
Acidic foods and drinks should not be consumed in conjunction with antibiotics, % (n)	38.64 (17)
Antifungals should be taken with fat-rich meals, % (n)	6.82 (3)
Propranolol should not be taken with protein-rich meals, % (n)	15.91 (7)
Broccoli, Brussels sprouts, and spinach should be avoided when taking Warfarin, % (n)	34.09 (15)
Patients with hypothyroidism should avoid Brussels sprouts, turnips, and cauliflower, % (n)	18.18 (8)
Patients taking Warfarin should avoid consuming broccoli, cauliflower, and chickpeas, % (n)	47.73 (21)
Timing of drug intake concerning food, % (n)	
Omeprazole before meals	68.18 (30)
Fibrates during meals	45.45 (20)
Metformin during or after meals	29.55 (13)
NSAIDs during or after meals	84.09 (37)
Levothyroxine before meals	54.55 (24)
Interactions between alcoholic beverages and the following drugs or therapeutic groups, % (n)	
Paracetamol	86.36 (38)
Oral contraceptives	63.64 (28)
Diclofenac	72.73 (32)
Benzodiazepines	75.00 (33)
Rupatadine	65.91 (29)
Antibiotics	84.09 (37)
Calcitriol	52.27 (23)

^a^ Only correct answers are shown. Data expressed as percentages (n). Abbreviations: MAOI, monoamine oxidase inhibitors; NSAIDs, non-steroidal anti-inflammatory drugs.

**Figure 1.**
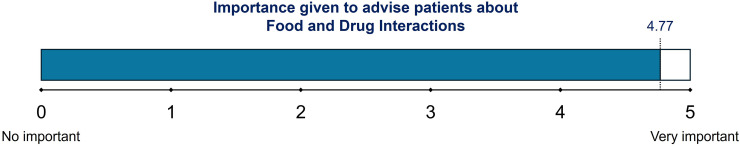
The importance given to advise patients about food and drug interactions by nutrition sciences and nutrition and dietetics students.

It was noted that most students did indeed believe that providing patients with advice on drug–food–drink interactions was essential, with an average importance rating of 4.77 on a scale of 0 to 5 (Figure 1). Finally, it was asked to the Nutrition Sciences students to perform a self-reflection about the importance and dissemination of information about FDIs (Table 5). The large majority of the students considered that the most appropriate way to increase knowledge about FDIs in the population was the guidance provided by the nutritionist during nutrition counseling, following the explanation given by the doctor when prescribing medication (86.36% and 84.09%, respectively). When asked about the importance of keeping up to date with FDIs to give correct advice to future patients and clients, the response was unanimous, and all the participants considered it essential. Regarding the awareness about FDIs, the majority of the participants (52.27%) considered that deepening knowledge and addressing the topic in different course subjects could be strategies to follow.

**Table 5.** Self-reflection on the dissemination of Food–Drug Interactions knowledge.

	Total sample (n = 44)
Most appropriate ways to increase the population's knowledge about FDIs, % (n)	
Read the information on the label and package leaflet of the medicine	63.64 (28)
Information leaflets with the most common interactions	73.73 (32)
Advice provided by the nutritionist during the appointment	86.36 (38)
Doctor's explanation when prescribing the medication	84.09 (37)
Pharmacist's explanation during the purchase of medication	54.55 (24)
All	29.55 (13)
Considered essential to stay updated on FDIs, to provide correct advice to future patients, % (n)	100.00 (44)
Awareness about FDIs can be improved, % (n)	
Develop a course/ university subject on the topic	18.18 (8)
Deepening knowledge/ addressing the topic in the different subjects	52.27 (23)
Conducting workshops and specific lectures on the subject	11.36 (5)
Taking a practical approach to FDIs through clinical cases	13.64 (6)
Did not know/ Not answered	4.55 (2)

Data expressed as percentages (n).

Abbreviations: FDIs, food–drug interactions.

## Discussion

To the best of our knowledge, this article was the first to assess FDIs knowledge among Portuguese Nutrition Sciences bachelor students. The present analysis concluded that there seems to exist a general lack of knowledge about FDIs in future Nutritionists, especially the possible mechanisms of interactions and in identifying specific examples of FDIs. Similar results were reported by Syed Snr et al. (2022) in a study conducted at King Saud University in Riyadh, Saudi Arabia. Using 12 questions, the author assessed the FDIs knowledge of 142 students and concluded that their level of knowledge was unsatisfactory. However, it is important to note that that study was carried out on pharmacy students, not nutrition students, as well as other existing scientific evidence carried out on health professionals, such as doctors, nurses, pharmacists, and nutritionists, but not on students or nutrition students (Benni et al., 2012; Coelho & Horta, 2021; Colet et al., 2016; Degefu et al., 2022; Osuala et al., 2021, 2022; Radwan et al., 2018; Silva et al., 2013; Zawiah et al., 2020). Nevertheless, all those articles showed similar conclusions, highlighting that the majority of these health professionals showed inadequate, low, and insufficient knowledge, revealing several gaps in this subject, particularly in identifying foods that have interactions with certain drugs, which once again confirms the results obtained in the present study.

Notwithstanding the evident lack of knowledge on the subject, among both health professionals and students, it can be seen that the participants in this study recognize the importance of knowing the topic to be able to advise their future patients and clients more effectively, thus optimizing their health and preventing possible complications arising from inadequate advice or medication use (Benni et al., 2012; Coelho & Horta, 2021; Colet et al., 2016; Degefu et al., 2022). The general lack of knowledge on this topic may be due to the small number of hours dedicated to this subject in nutrition courses, the insufficient interdisciplinarity where this subject could be covered in various curricular units, or other related pedagogical approaches. FDIs can have relevant clinical implications for patients; therefore, it would be interesting to approach this subject from a more practical and real perspective, including clinical cases, besides the main theoretical concepts. On the other side, food is a complex matrix containing several nutrients and different compounds that are capable of interacting with drugs, which makes it more difficult to study. Furthermore, there are several differences in the bioavailability of drugs in patients that depend on many factors beyond diet (e.g. age, illnesses, and other drugs), which also contributes to a lack of knowledge in this field.

In this sense, when asked how awareness of FDIs could be improved, the participants in this study recognized the importance of more comprehensive training, particularly during their undergraduate degree. Conversely, when the same question was posed to nurses participating in Enwerem & Okunji's study to assess knowledge, attitudes, and awareness of FDIs interactions, the participants suggested that its preferred way was a specific 6-monthly training sessions (Enwerem & Okunji, 2015). This suggestion underlines the need to be constantly up to date about FDIs, as confirmed by the students in this research since all participants considered that it is essential to provide adequate guidance to their future patients. Another way to improve this knowledge can also be achieved through strategies such as reviewing the Nutrition Sciences bachelor curriculum, and/or by implementing dynamics that promote interpersonal knowledge sharing, involving, for example, pharmacy and nutrition students. These were the main conclusions reached by Almazrou & Alaujan (2022), whose research demonstrated the effectiveness of this learning method in transmitting FDIs knowledge among students (Almazrou & Alaujan, 2022).

Improving nutritionists’ knowledge as well as their interest in this subject has the potential to significantly benefit society, making a valuable contribution to promoting the population's health and well-being. This relationship is because FDIs play a fundamental role in the success of therapeutic treatments and can considerably influence both quality of life and patient safety.

The main strengths of this study reside mainly in the use of an adapted version of an FDIs-specific and validated questionnaire (Benni et al., 2012), and in the fact that this preliminary evaluation can also be used as a model by other academic institutions, both national and international, in a way to improve knowledge of these future professionals, according to the gaps identified. However, it is also important to recognize some limitations, such as that these results were self-reported through an online questionnaire and without a time limit for answering. This implies possible social desirability errors, such as the possibility that only students interested in this subject participated. Additionally, the small sample size (in the number of students and universities) as also the cross-sectional design makes these results not representative and not possible to establish a cause–effect relationship, respectively. The fact that most of the participants were from the same university does not allow us to affirm if differences in the curricular plan might influence the knowledge about FDIs, and this sample heterogeneity should also be assumed. Conversely, this research is presented as a pilot study and, with some adaptations, could be expanded to a more comprehensive scale.

Hence, FDIs have significant implications for the education and clinical practice of nutritionists. Regarding education, nutrition courses’ curricular plans must include comprehensive education on pharmacology, covering FDIs main concepts, examples, mechanisms, and their clinical implications. The number of hours assigned to this topic should be increased, and ideally, it should be covered in more than one curricular unit or in a specific one. Additionally, nutrition students should have interdisciplinary training, learning closely with pharmacists, medical doctors, and other healthcare professionals. This can be achieved with the presence of different health professionals in the classes and by addressing not only theoretical topics but also specific clinical cases, leading to better patient care and medication management. Moreover, students, as future nutritionists must pursue continuous education, to stay updated on new drugs and recent research about FDIs. Regarding the clinical practice of nutritionists, due to their intervention in patients’ dietary habits and diets, it is crucial that they better predict and manage potential FDIs. Nutritionists based on their knowledge of FDIs can create personalized nutrition plans that minimize or avoid interactions and monitor and identify some symptoms associated with FDIs. Additionally, nutritionists must have a key role in educating patients about FDIs, including foods to avoid, understanding labels, and recognizing the importance of patient's adherence to both diet and medication.

In conclusion, the main findings of this study shed light on the current state of knowledge and awareness among Nutrition Sciences and Nutrition and Dietetics students regarding FDIs. In summary, a general lack of knowledge about FDIs among Portuguese students of Nutrition Sciences Bachelor was observed, especially in identifying and understanding specific interactions and appropriate dietary management. Nevertheless, the participants recognized the importance of the Nutritionist as a health professional as well as the need for more knowledge, on this topic to provide correct advice to their future patients. Addressing these gaps through targeted educational interventions and interdisciplinary collaboration is crucial to better prepare (future) nutrition professionals to effectively mitigate the risks associated with FDIs and optimize patient outcomes in clinical practice.
